# The effect of combining punishment and reward can transfer to opposite motor learning

**DOI:** 10.1371/journal.pone.0282028

**Published:** 2023-04-10

**Authors:** Cong Yin, Tian Gao, Biao Li

**Affiliations:** Capital University of Physical Education and Sports, Beijing, China; The Ohio State University, UNITED STATES

## Abstract

Recent laboratory findings have demonstrated that, when imposed separately, punishment and reward have different effects on motor learning. In real-world applications, however, they are usually used in combination to improve human behavior. For instance, a student may be punished when failing an examination and rewarded when getting a high score. It remains unclear precisely how people are motivated when punishment and reward are combined. Moreover, whether it is possible for the effects of punishment and reward to transfer to other learning situations remains unknown. In the present study, four groups of participants were trained on a motor adaptation task under conditions of either punishment, reward, both punishment and reward combination, or a neutral control condition (neither). We tested what the effect of combining punishment and reward is on motor learning and memory. Further, we examined whether the effect could transfer to later opposite-direction learning in the absence of motivational feedback. Specifically, during the initial learning when there is motivational feedback, combining punishment and reward can not only accelerate learning rate, but can also increase learning extent. More interestingly, the effect can even transfer to later opposite-direction learning. The findings suggest that the combination of punishment and reward has a distinct advantage over pure punishment or reward on motor learning and the effect can transfer to opposite motor learning.

## Introduction

It has long been established that punishment and reward are potent modulators of human and animal behavior [1–3]. For instance, out of fear of being punished for failing the examination, students work hard. Eager to receive a scholarship to pay for college or university, they work even harder. However, until only recently, the ways in which punishment and reward specifically influence human motor learning have not been extensively studied. The limited previous research that has been conducted has shown that negative and positive feedback have dissociable effects on motor learning. For a procedural learning task, punishment is found to be associated only with improvement in performance when there was no sequence to be learned while reward enhances implicit learning of the motor sequence [4]. For a motor skill learning task, reward improves long-term motor memory retention while punishment does not [5]. For a motor adaptation task, punishment is related to faster learning while reward increases retention [6].

Motivating behavior in humans often employs a technique proverbially named for motivating animal behavior, the “carrot-and-stick” approach: to persuade a donkey or other draft animal to move, one can either strike it with a stick, or dangle a carrot just out of its reach [2]. In reality, combining negative and positive feedback together seems on its face to be a more straightforward and efficient way to motivate: mistakes are met with punishment, while correct behavior is met with reward. As diverse as human behavior is, it is unreasonable to expect either exclusively making mistakes or perfectly correct behavior, and as such, humans in the real world would receive neither exclusively punishment nor exclusively reward. As the example mentioned above, the students have both the chances to get punishment and reward according to their academic achievement. However, aforementioned studies of feedback and performance used experimental designs that examined effects of positive, negative, or neutral feedback in isolation, leaving unanswered the question of whether combining punishment and reward would offer advantages in combination or show distinct effects on motor learning and memory. In addition, for practical applications such as sports training and motor rehabilitation, motor learning is more useful if it successfully transfers to untrained scenarios. Previous studies indicate that the effects of punishment [6] and reward [5] can last until people relearn. However, it is not clear whether the effect of punishment and reward could transfer to other situations when motivational feedback is no longer provided.

Motor adaptation, the way in which humans make quick adjustments to motor performance in altered conditions, has served as a cornerstone in understanding motor learning [7]. In the present study, we used a motor adaptation task to answer the abovementioned questions: first, what the effect of combining punishment and reward is on motor learning and memory, and second, whether the effect could transfer to other situations. Specifically, we recruited four groups of participants who initially learned a visuomotor rotation task under pure punishment, pure reward, punishment and reward combination, or neutral control conditions, during which we tested the motivational effect on motor learning. We then tested effects on memory retention and consolidation after initial learning when motivational feedback was no longer provided. In addition, without any motivational feedback, we tested whether negative or positive feedback on initial learning could transfer to later learning a visuomotor rotation task in the opposite direction. We found a distinct effect of combining punishment and reward on motor learning comparing to the summation of their separate effect. More interestingly, the effect of combining punishment and reward could transfer to the following opposite-direction learning. Our findings provide new insights into the mechanisms underlying motivational effect on motor learning, and may have important implications for practical applications such as sports training and motor rehabilitation.

## Methods

### Participants

60 right-handed participants were recruited for the experiment (41 men; average age = 20.5 ± 1.6 yr; n = 15 for each of the four experimental groups). All participants were naïve to the purpose of the study, signed an institution-approved consent form, and were paid to participate. All experimental procedures were approved by the Institutional Review Board of Capital University of Physical Education and Sports.

### Experimental setup

**Fig 1:**
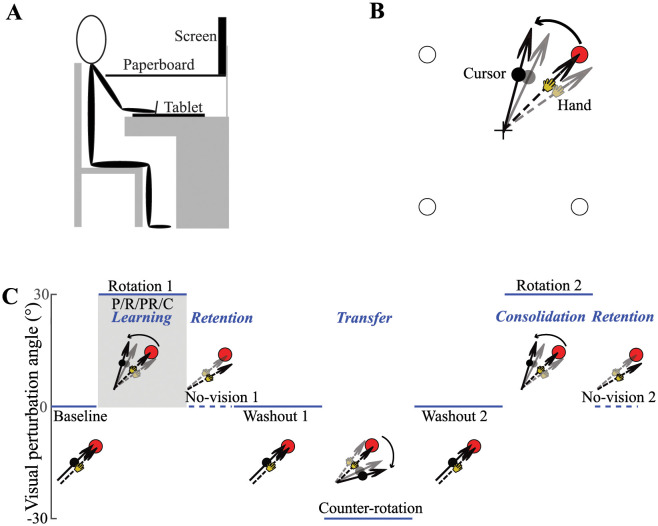
Experimental setup and designs. A. Side view of the experimental setup. B. The illustration of the four possible targets and the perturbation of CCW visuomotor rotation. When the participant moves their hand to the top-right target (the dashed arrow), the cursor represented by the black circle was rotated 30° CCW to the hand position (the solid arrow). Then the hand gradually rotates clockwise over trials to bring the cursor to land on the target (the grey arrows). C. Eight phases of trials: baseline, initial rotation learning, initial no-vision, initial washout, counter-rotation, second washout, relearning, and second no-vision. The positive angle indicates CCW rotation. The dashed horizontal lines during no-vision phases indicate no visual feedback. The dashed and solid arrows denote the trajectory of hand and cursor movement, respectively. Shaded gray: participants received punishment (P), reward (R), combination of punishment and reward (PR) or random number (C) during initial rotation learning phase.

Each participant sat in a height-adjustable chair facing an LCD monitor, mounted vertically in front of them at eye level. The monitor had a 27-inch screen with a refreshing rate of 60 Hz. A digitizing tablet (Wacom PTH 860) was placed on the horizontal desktop before the seating participant. A black paperboard placed horizontally underneath the monitor occluded vision of the hand. Each participant used the right hand to hold a digitizing pen whose position was measured in real time by the tablet with a resolution of 0.005 mm and a sampling frequency of 200 Hz (Fig 1A). The data acquisition codes were written in MATLAB (MathWorks).

### Basic movements

Participants were instructed to make rapid center-out shooting movements to four radially-arranged targets (white discs, 8 pixels in diameter) placed on a 5 cm-radius circle with an angular separation of 90°. The task required participants to use a hand-controlled cursor to slide through the target as accurately as possible. Their hand position was measured by the digitizing pen and presented as a cursor (a green disc, 4 pixels in diameter) when needed. At the beginning of each trial, a participant rested the digitizing pen on a 4-mm plastic disc glued onto the tablet. The disc was also used as an anchor to guide the participant to return the unseen finger to the starting position after each trial. A visual starting position, depicted as a yellow cross (4 pixels per line), was overlaid on the plastic disc. The hand cursor was only visible within 4 mm around the starting position. Once the finger had remained at the starting point for 100 ms, a target would appear, and a computer speaker produced a beep to signal the participant to move. The feedback cursor was visible until the movement amplitude exceeded 5 cm. A beep sound was also played to signal the participant to bring the finger back to the starting position for the next trial once he/she stopped the movement. A low-pitched tone warned the participant when they reacted and moved too fast or too slow (reaction and movement time < 200 ms or > 800 ms). Before formal data collection, the participant practiced a few unperturbed trials to familiarize himself/herself with the required movement speed.

### Experimental procedures

We sought to explore the effect of combining punishment and reward on motor learning and memory. Further, we tested whether the effect could transfer to other learning situations. To achieve the two aims, participants were asked to learn a visuomotor rotation task using task A → task B → task A paradigm [8] with similar procedures. Participants except for the *Control* group (see below) were given motivational feedback only in the initial learning of task A, during which the effect on motor learning was tested. Motivational effect on memory retention was tested immediately after learning of task A when there was neither cursor feedback, nor motivational feedback [6, 9]. The effect on motor consolidation, which was defined as resistance to retrograde interference (interference by task B on initial learning of task A) was tested on relearning of task A [8]. Whether the motivational effect can transfer to other learning situations was tested during task B. Specifically, we divided the experiment session into eight consecutive phases: baseline, initial rotation learning (task A), initial no-vision, initial washout, counter-rotation learning (task B), second washout, relearning (task A), and second no-vision. In the baseline phase, participants received veridical cursor feedback for twenty 4-trial cycles, each cycle requiring participants to move to one of the four targets once. This helped participants become familiar with the reaching task and served to compute each participant’s direction biases associated with moving to different targets. In the initial learning phase, participants experienced an abrupt perturbation when the hand cursor was rotated 30° counterclockwise (CCW) and remained rotated for 40 cycles. Participants were expected to move their hands clockwise (CW) to counter the CCW rotation perturbation (Fig 1B). Only during this phase, every group except for the *Control* group received different additional motivational feedback depending on the experimental condition (except for this phase, the task was identical across the four groups). In the initial no-vision phase, participants were given neither motivational feedback, nor cursor feedback, and were instructed to move directly to the target without using any strategy for 20 cycles. The aftereffects during this phase served to measure the motivational effect on memory retention. In the initial washout phase, participants were given veridical cursor feedback for 40 cycles, which served to remove the potential anterograde interference by Task A on subsequent learning of Task B. In the counter-rotation learning phase, participants experienced an abrupt perturbation for 40 cycles, but this time the hand cursor was rotated 30° CW which was contrary to the initial learning phase. This served to explore whether any motivational effects on task A could transfer to learning on Task B. In the second washout phase, participants again experienced veridical feedback for 40 cycles to washout the apparent effect of counter-rotation learning. In the relearning phase, participants again experienced a 30° CCW rotation perturbation for 40 cycles. This served to examine the motivational effect on motor consolidation. Finally, in the second no-vision phase, participants were again given neither cursor feedback, nor motivational feedback, and instructed to move directly to the targets without any strategy for 20 cycles. This served to measure the memory retention after relearning of Task A. An illustration of the experimental designs is shown in Fig 1C. Participants received pertinent instructions prior to the start of each phase. Each phase was separated by a short rest period (<1 min) in which participants were instructed to remain on the chair.

Participants were divided into four groups according to the different types of feedback they got during the initial learning phase. For the *Punishment* group, a score ranging from −10 to 0 was presented 1 cm above the target as soon as the participants reached to the invisible 5 cm-radius circle on each trial. Participants began with 0 point and the points accumulated across the block. The punishment score (P) is the linear function of the absolute value of the directional error (*θ*): P = −2 * |*θ*| / 9 if |*θ*| ≤ 45°, P = −10 if |*θ*| > 45°. For the *Reward* group, the reward score (R) ranged from 0 to 10: R = −2 * |*θ*| / 9 + 10 if |*θ*| ≤ 45°, R = 0 if |*θ*| > 45°. For the *Punish and reward combination* (*PR*) group, participants could receive either positive or negative scores, ranging from −10 to 10 according to their performance: PR = −4*|*θ*| / 9 + 10 if |*θ*| ≤ 45°, PR = −10 if |*θ*| > 45°. For these three groups, participants were informed of the range of the score and that the fewer directional errors they make, the higher their payments would be. Participants in the *Punishment* group began with 60 yuan with 0.025 deduced per negative point, those in the *Reward* group began with 20 yuan with 0.025 earned per positive point, and those in the *PR* group began with 40 yuan with 0.025 earned for one positive point and deduced 0.025 for one negative point. To control for the potential distractor effect of the changing scores in the motivational groups, for the *Control* group, a random number ranging from -10 to 10 was presented at the same position as other groups. Participants in the *Control* group were informed that the number had no relationship to their performance and were instructed to ignore it. They were given a fixed participation fee.

### Data analysis

The direction of the shooting movement indicated the participants’ adaptive response to the visuomotor rotation, which was determined by computing the direction of the vector spanning from the starting position to the position of the hand at the peak outward velocity. We computed the relative angle (abbrev. angle) between the movement direction and the target direction. A positive angle indicates a clockwise rotation that compensates the CCW rotation perturbation. We also computed each participant’s direction biases associated with moving to different targets based on the mean of the last 10 cycles in the baseline phase. These direction-specific baseline biases were subtracted from the movement directions obtained in later learning phases.

During the initial learning phase, all four groups reduced their directional errors rapidly within the first half of the cycles. We thus used the window of the first 20 cycles to compute the learning rates of the initial learning, counter-rotation learning, and relearning phases. The learning rate was operationally defined as the average angle within the window (for similar treatments, see [10, 11]). Of note, our main results are insensitive to the choice of learning rate window: using a window size between the first 15 to 30 cycles did not produce different inferential statistics. The difference of the learning rates between the initial learning phase and the relearning phase served as a measure of savings (i.e., faster relearning rate). We used the final 10 cycles for each learning phase to calculate the learning extent and used all learning cycles to reflect the whole learning. Learning rate, learning extent, and whole learning are three important indicators used to depict participants’ motor learning. Learning rate reflects how fast participants adapt to the new perturbation during early learning. Learning extent reflects how well participants learn the task during late learning. Whole learning reflects overall learning throughout a learning phase. During the two no-vision phases, we used all the cycles to reflect memory retention. One-way ANOVA was used to compare the performance among the four groups for each phase. The Tukey honest significant difference (HSD) test was used for multiple post hoc comparisons. Assumptions of normality were examined prior to conducting *t*-tests and ANOVA, and all dependent variables satisfied the assumption. Prior to statistical analyses, we excluded trials in which the movement direction was more than three standard deviations from the mean for that cycle. This resulted in an average removal of less than 0.5% of the trials per participant. The significance level was set at α = 0.05. All analyses were performed in SPSS 26.0. Before focusing on the motivational effects of combining punishment and reward, we first examined the overall behavior of the *Control* group across all phases.

## Results

### The *Control* group

**Fig 2:**
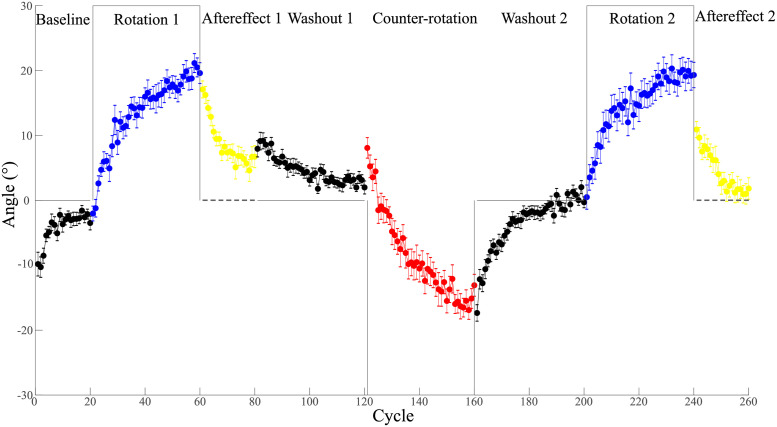
Learning data for all the 8 phases for the *Control* group. The grey lines denote the perfect hand movement direction to counter the visual perturbation. The dashed lines denote no visual cursor feedback. Positive angle denotes CW direction. Error bars denote SEM.

The *Control* group exhibited a typical interference pattern in the ABA paradigm as previously reported [8, 12]: they performed worse when learning counter-rotation than initial rotation and showed no savings during relearning compared to initial learning (Fig 2). Specifically, during the initial learning phase, they learned the rotation rapidly during the first half and the learning rate, defined as the average angle over the first half of the learning cycles, was 9.24 ± 0.92° (mean ± standard deviation, same below). The participants kept learning for the second half of the cycles to an extent of 18.95 ± 1.37°. Then during the initial no-vision phase, the rotation memory quickly decayed from 17.10 ± 1.30° to 6.03 ± 1.33° and kept steady during the last 5 cycles. For the initial washout phase when the veridical feedback was provided, participants seemed to firstly recall the rotation memory for the first 6 cycles and later gradually returned to near baseline levels (1.99 ± 1.00° for the last cycle). During the counter-rotation phase, participants again first recalled the rotation memory and then persisted in trying to adapt to the opposite rotation perturbation. The counter-rotation learning rate was only 3.70 ± 1.58°, which was significantly slower than the initial learning rate (paired *t*-test, *t*_(14)_ = −4.13, *p* < 0.001, *d* = 1.08). By the end of this phase, participants achieved to a learning extent of 15.09 ± 1.46°, which was significantly less than that of initial rotation learning (paired *t*-test, *t*_(14)_ = −3.26, *p* = 0.006, *d* = 0.84). This indicates an apparent anterograde interference effect by initial rotation on counter-rotation learning [12]. The second washout phase again brought participants from levels seen during counter-rotation to those seen during baseline. For the following relearning phase, the learning rate was 11.11 ± 1.86°, which showed no savings when comparing to the learning rate of initial rotation learning (paired *t*-test, *t*_(14)_ = 1.06, *p* = 0.31, *d* = 0.27). This suggests a retrograde interference by counter rotation learning on initial learning [8]. Finally, for the second no-vision phase, participants started from a significantly lower retention level (10.93 ± 1.24°, paired *t*-test, *t*_(14)_ = −3.35, *p* = 0.005, *d* = 0.87) than the first no-vision phase and drifted to 1.48 ± 1.49°, which was also lower than initial retention during the first no-vision phase (last 5 cycles, paired *t*-test, *t*_(14)_ = −2.83, *p* = 0.013, *d* = 0.73).

### Initial rotation learning

**Fig 3:**
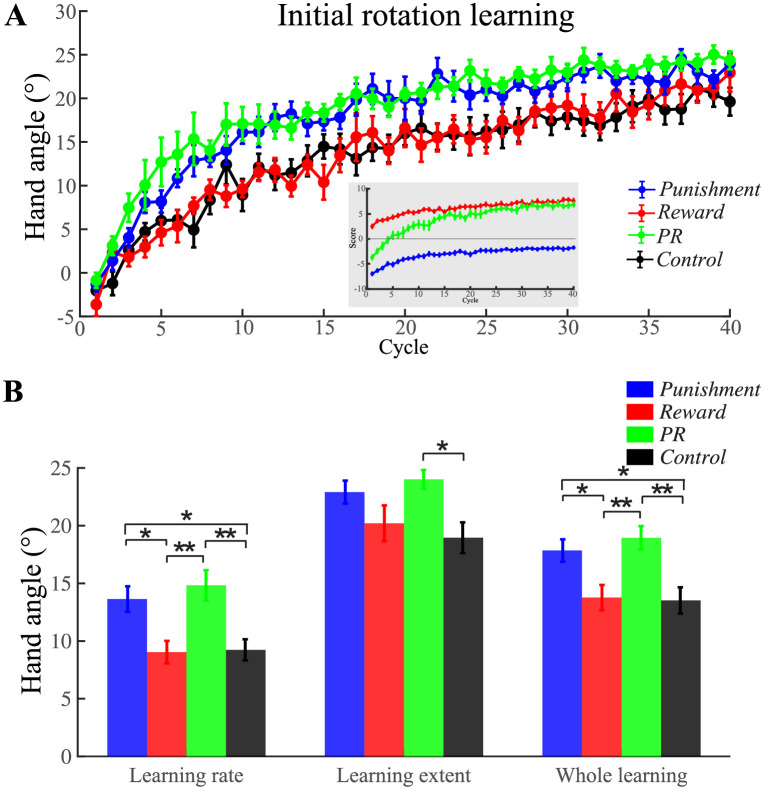
Comparison of the learning during the initial rotation learning phase from four different groups. A) The learning curves during the initial rotation learning phase of the four groups. Inset illustrates the score curves for the three motivational groups. B) Average learning rate, average learning extent, and average hand movement direction of the whole learning cycles of each group. Positive angle denotes CW direction. Error bars denote SEM. *p < 0.05, **p < 0.01.

Before examining motivational effects on motor learning, we first confirmed that the four groups exhibited no significant difference during the baseline phase. Fig 3A shows the learning curves of the four groups during initial rotation learning. Of note, three motivational groups received score feedback according to their performance only during this phase. The score increased from -6.95 ± 0.21, 2.5 ± 0.30, and -3.68 ± 0.38 (the first cycle) to -1.733 ± 0.17, 7.56 ± 0.42, and 6.86 ± 0.37 (the last cycle), for the *Punishment*, *Reward* and *PR* groups, respectively. We sought to explore whether different types of feedback would influence people’s motor learning during this phase. The learning rate for *Punishment*, *Reward*, *PR*, and *Control* group was 13.64 ± 1.10°, 9.04 ± 0.98°, 14.82 ± 1.32°, and 9.24 ± 0.92°, respectively (Fig 3B). One-way ANOVA of learning rates showed a significant group difference (*F*_3, 56_ = 7.47, *p* < 0.001, $\eta_{p}^{2}=0.29$, Fig 3B). Tukey HSD post-hoc pairwise comparisons showed the *Punishment* and *PR* groups learned significantly faster than both the *Control* (mean difference = 4.40° and 5.59°, *p* = 0.03 and 0.003) and *Reward* groups (mean difference = 4.60° and 5.79°, *p* = 0.02 and 0.002). The learning extent of the four groups was 22.91 ± 0.93°, 20.20 ± 1.59°, 24.01 ± 0.76°, and 18.95 ± 1.37°, respectively. One-way ANOVA of learning extent showed a significant group difference (*F*_3, 56_ = 3.73, *p* = 0.02, $\eta_{p}^{2}=0.17$). Tukey HSD post-hoc pairwise comparisons suggest that the *PR* group learned more than the *Control* group at the end of the initial rotation learning phase (mean difference = 5.05°, *p* = 0.02). For the whole learning session, the average learning amount of all the 40 learning cycles for the four groups was 17.85 ± 0.97°, 13.76 ± 1.10°, 18.94 ± 1.02°, and 13.52 ± 1.14°, respectively. One-way ANOVA also showed a significant group difference (*F*_3, 56_ = 6.90, *p* < 0.001, $\eta_{p}^{2}=0.27$) of the whole learning. Tukey HSD post-hoc pairwise comparisons again showed greater learning by the *Punishment* and *PR* groups over the *Control* (mean difference = 4.32° and 5.42°, *p* = 0.03 and 0.003) and *Reward* groups (mean difference = 4.08° and 5.18°, *p* = 0.04 and 0.006). Therefore, the data during the initial rotation learning phase shows that both *Punishment* and *PR* groups learned more overall at a faster rate than *Control* and *Reward* groups. Moreover, the *PR* group learned more completely during later stages.

### Initial no-vision and initial washout phase

For the initial no-vision phase, during which the motivational effect on memory retention was tested, neither the performance of the first cycle (*F*_3, 56_ = 0.69, *p* = 0.56, $\eta_{p}^{2}=0.04$) nor the average of the last 5 cycles (*F*_3, 56_ = 0.97, *p* = 0.42, $\eta_{p}^{2}=0.05$) showed any significant difference among the four groups. This tendency was further confirmed by the average performance of the whole phase (*F*_3, 56_ = 1.01, *p* = 0.40, $\eta_{p}^{2}=0.05$), indicating that different kinds of feedback did not make a difference on memory retention.

During the initial washout phase when the visual feedback was provided again, all the participants were reminded of the rotation memory to some degree. The average hand movement direction of the first three cycles for *Punishment*, *Reward*, *PR*, and *Control* group was 10.29 ± 1.07°, 7.61 ± 0.83°, 11.59 ± 1.04°, and 8.75 ± 1.16°, respectively. One-way ANOVA also showed a significant group difference (*F*_3, 56_ = 2.84, *p* = 0.046, $\eta_{p}^{2}=0.13$). Tukey HSD post-hoc pairwise comparisons indicated *PR* brought back more rotation memory than *Reward* group. After that, the rotation memory was washed out gradually by the end of the phase. The average hand movement direction of the last 5 cycles for *Punishment*, *Reward*, *PR*, and *Control* group was 2.33 ± 0.54°, 2.81 ± 0.59°, 2.69 ± 0.61°, and 2.77 ± 0.66°, respectively. No significant group difference was found among the four groups (*F*_3, 56_ = 0.13, *p* = 0.94, $\eta_{p}^{2}=0.01$). This suggests that the greater learning when combining punishment and reward during the initial learning phase was extended to the early initial washout phase.

### Counter-rotation learning phase

**Fig 4:**
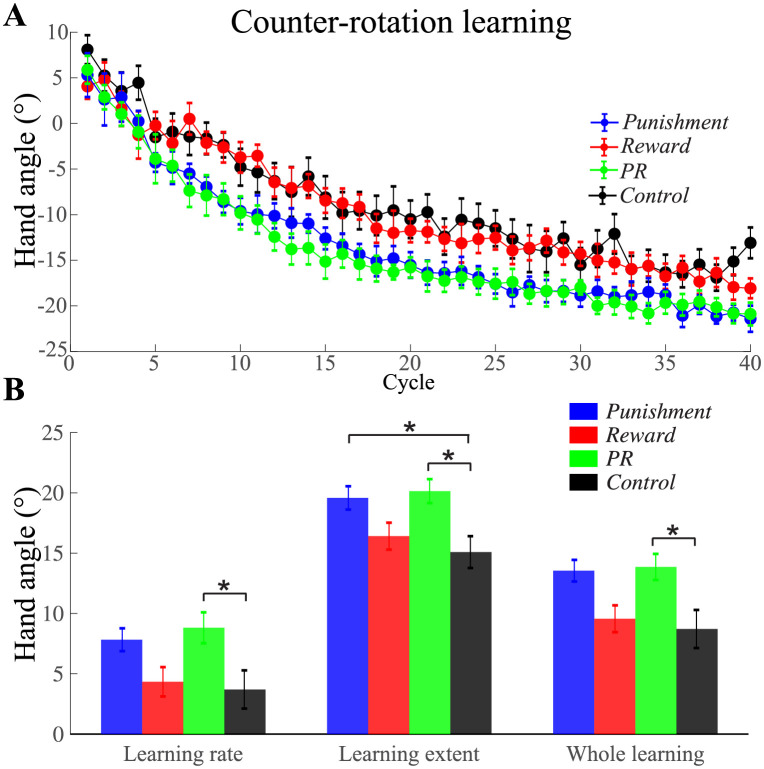
Comparison of the learning during the counter-rotation phase for the four groups. A) The learning curves in the counter rotation phase of the four groups. B) Average learning rate, average learning extent, and average hand movement direction of the whole learning cycles of each group. Note the opposite number of participants’ actual hand movement direction is calculated here. Positive angle denotes CW direction. Error bars denote SEM. *p < 0.05.

Whether motivational effects could transfer to opposite-direction learning was tested during the counter-rotation learning phase. At the beginning of this phase, participants first attempted the same movement solution they learned to counter the CCW rotation they previously encountered. Upon finding it was not suitable for the current situation, they gradually moved to the opposite direction to adapt to the opposite rotation. Although they received the same kind of feedback during the counter-rotation phase, different groups of participants exhibited different learning (Fig 4A). For the convenience of comparison with the two rotation learning phases, we first calculated the opposite number of their hand movement angle to do further analysis. The learning rate of *Punishment*, *Reward*, *PR*, and *Control* group was 7.83 ± 0.95°, 4.33 ± 1.22°, 8.81 ± 1.28°, and 3.70 ± 1.58° respectively (Fig 4B). One-way ANOVA revealed a significant group difference (*F*_3, 56_ = 3.92, *p* = 0.01, $\eta_{p}^{2}=0.17$) of the learning rate. Only one pairwise comparison reached significance: *PR* learned faster than *Control* group (mean difference = 5.12°, *p* = 0.03). The learning extent of the four groups was 19.58 ± 1.02°, 16.41 ± 1.13°, 20.14 ± 0.91°, and 15.09 ± 1.46°, respectively. One-way ANOVA revealed a significant group difference (*F*_3, 56_ = 4.52, *p* = 0.007, $\eta_{p}^{2}=0.20$) of the learning extent. Tukey HSD post-hoc pairwise comparison showed *Punishment* and *PR* learned to a larger extent than the *Control* group (mean difference = 4.49° and 5.05°, *p* = 0.04 and 0.02). Across all the learning cycles, the average learning amount of the four groups was 13.00 ± 0.85°, 9.56 ± 1.11°, 13.55 ± 0.90°, and 8.71 ± 1.58°, respectively. One-way ANOVA revealed a significant group difference (*F*_3, 56_ = 4.48, *p* = 0.007, $\eta_{p}^{2}=0.19$) of the entire learning phase. Tukey HSD post-hoc pairwise comparisons confirmed the *PR* group showed greater opposite-direction learning than *Control* group (mean difference = 4.84°, *p* = 0.02). Hence, these results suggest that the learning advantage of combining punishment and reward transfers from initial rotation learning to counter-rotation learning.

### Second washout phase

For the second washout phase, at first *Punishment* group kept more memory from the counter- rotation direction. The average hand movement direction of the first two cycles for the four groups was −18.82 ± 0.53°, −15.55 ± 1.19°, −18.39 ± 0.92°, and −14.76 ± 1.24°, respectively. One-way ANOVA showed a significant group difference (*F*_3, 56_ = 4.02, *p* = 0.01, $\eta_{p}^{2}=0.18$). Tukey HSD post-hoc pairwise comparisons showed that the *Punishment* group started from a more counterclockwise direction than the *Control* group (mean difference = 4.06°, *p* = 0.03). However, this tendency did not last for later cycles. The average hand movement direction of the last five cycles for the four groups was −1.27 ± 0.58°, −1.95 ± 0.76°, −0.48 ± 0.48°, and 0.79 ± 0.73°, respectively. One-way ANOVA showed a significant group difference (*F*_3, 56_ = 3.31, *p* = 0.03, $\eta_{p}^{2}=0.15$). Tukey HSD post-hoc pairwise comparisons revealed that *Control* group moved more to the clockwise direction than *Reward* group (mean difference = 4.06°, *p* = 0.02). Although some differences among the groups, they showed no significant difference for the average of the whole cycles (*F*_3, 56_ = 0.94, *p* = 0.43, $\eta_{p}^{2}=0.05$).

### Relearning phase

**Fig 5:**
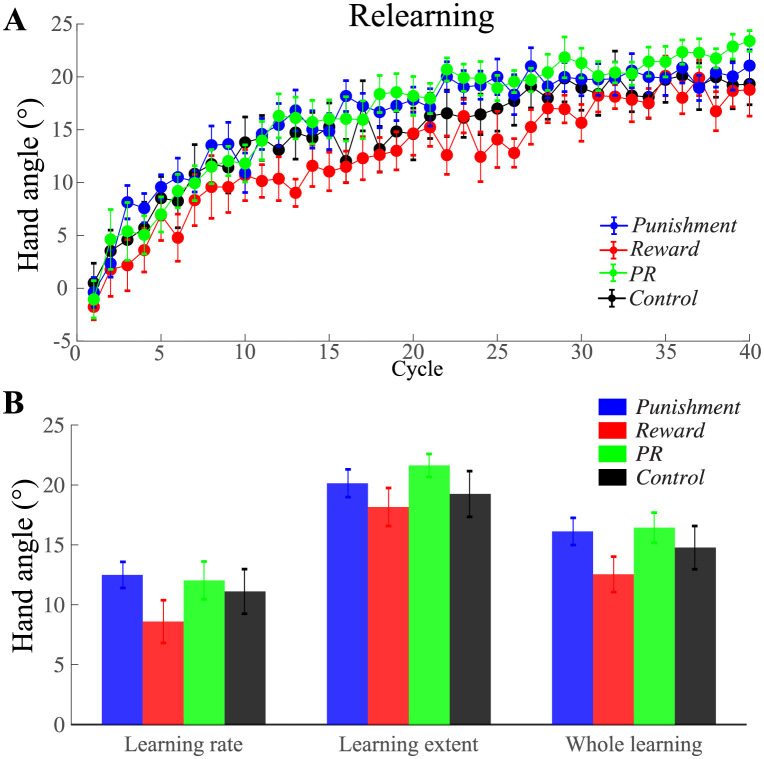
Comparison of the learning during the relearning phase from the four groups. A) The learning curves in the relearning phase of the four groups. B) Average learning rate, average learning extent, and average hand movement direction of the whole learning cycles for each group. Positive angle denotes CW direction. Error bars denote SEM.

Fig 5A shows the learning tendency of the four groups during the relearning phase, during which the motivational effect on memory consolidation was tested. The learning rate for *Punishment*, *Reward*, *PR*, and *Control* group was 12.49 ± 1.09°, 8.60 ± 1.79°, 12.03 ± 1.58°, and 11.11 ± 1.86° respectively (Fig 5B). One-way ANOVA showed no significant group difference (*F*_3, 56_ = 1.17, *p* = 0.33, $\eta_{p}^{2}=0.06$). The learning extent of the four groups was 20.14 ± 1.26°, 18.16 ± 1.40°, 21.63 ± 0.97°, and 19.25 ± 1.83° respectively. No significant difference was found among groups (*F*_3, 56_ = 1.10, *p* = 0.36, $\eta_{p}^{2}=0.06$). The average reaching direction for the whole phase was 16.12 ± 1.13°, 12.54 ± 1.48°, 16.43 ± 1.26°, and 14.77 ± 1.80° respectively. Also, no significant difference was found among groups (*F*_3, 56_ = 1.51, *p* = 0.22, $\eta_{p}^{2}=0.08$), indicating the learning difference caused by different kinds of feedback did not extend to the relearning phase. That is, motivational feedback did not make a difference to memory consolidation.

### Second no-vision phase

Finally, for the second no-vision phase, during which the memory retention was tested again, the average reaching direction for the first cycle for *Punishment*, *Reward*, *PR*, and *Control* group was 14.00 ± 1.39°, 13.40 ± 1.06°, 14.16 ± 1.10°, and 10.93 ± 1.24° respectively. One-way ANOVA showed no significant group differences (*F*_3, 56_ = 1.55, *p* = 0.21, $\eta_{p}^{2}=0.08$). The average reaching angle for the last five cycles for the four groups was 7.12 ± 3.05°, 5.08 ± 2.08°, 5.88 ± 1.82°, and 1.45 ± 1.49° respectively. No significant group difference (*F*_3, 56_ = 1.24, *p* = 0.30, $\eta_{p}^{2}=0.06$) was found. The average reaching angle during the whole phase for the four groups was 8.74 ± 2.43°, 7.71 ± 1.72°, 7.94 ± 1.67°, and 4.41 ± 1.36° respectively. No significant group difference (*F*_3, 56_ = 1.08, *p* = 0.36, $\eta_{p}^{2}=0.06$) was found. This replicated the results observed for the initial no-vision phase showing that different kinds of feedback did not make a difference on memory retention.

## Discussion

Findings from our study demonstrate a unique capability of combining punishment and reward: not only does it have the potential to accelerate learning rate, but could also enhance learning extent. More interestingly, the effect of combining punishment and reward could even transfer to later opposite rotation learning.

Previous studies investigating motivational effects on motor learning sought to dissociate the effects of negative and positive feedback [4–6, 9, 13–16]. Researchers tried to compare the different effects between punishment and reward, and further explore their respective neural bases. However, in the real world, punishment and reward are used in combination in the majority of cases. For example, at school, a student might face punishment if he does not finish his homework, but might later be praised if he helps another classmate or raises a valuable question. Only in rare real-world situations would someone receive exclusively punishment or reward. The sole use of either punishment or reward has limitations in both cases: always using punishment will bring about fear and hostility, while always using reward will cause complacence and arrogance [17]. Therefore, it is essential to investigate the combined effect of punishment and reward on motor learning, instead of studying them separately.

During initial rotation learning phase, different from pure punishment or pure reward, combining punishment and reward could not only make people learn faster at first, but learn more completely during later stages. That is, the learning advantage of combining punishment and reward lasts for the entire learning session. Our results indicate that reinforcement by punishment and reward in combination is more effective than either punishment or reward alone. For the Punishment and Reward combination (*PR*) group in our design, the participants first encountered a large perturbation and showed large errors at first. Correspondingly, they received negative points during earlier stages, which is similar to the *Punishment* group. As suggested by previous research, negative feedback may increase people’s sensitivity to prediction errors [6]. In addition, people exhibit loss aversion in face of punishment [18, 19]. In order to avoid losses, they have more motivation to explore more directions [20, 21], thus finding a way to reduce directional error both more easily and more quickly. When the *PR* combination group reduced error to around 20°, however, the participants began to receive positive score (unlike the *Punishment* group). In order to receive more money, they persisted in applying the strategy they just used to reduce errors and found that it still worked. The result of continuing to reduce errors while earning more money motivated the *PR* combination participants to learn more completely during the later stages. On the contrary, because the *Reward* group received a reward from the very beginning, they might have less willingness to refine their movement direction even when achieving a relatively high score, according to the win-stay/lose-shift strategy [21]. Hence, it is possible that the continuous motivational effect of combining punishment and reward served to underpin the unique learning advantage of the *PR* group.

More interestingly, the learning advantage of combining punishment and reward transfers from initial learning to later opposite-rotation learning. It is not surprising that the faster learning rate from punishment could transfer from initial rotation learning to the subsequent relearning of the same rotation [6]. However, it is not known whether the motivational effect could transfer to a new learning context, especially to an extreme case, the opposite-rotation learning, as it has long been known that learning a given rotation will interfere with any immediately subsequent learning of a rotation of the opposite direction [7]. In our study, only *PR* group showed faster and better counter-rotation learning than the *Control* group. That is, only the effect of combining punishment and reward transferred from initial rotation learning to later opposite-direction learning. The faster learning rate and the better whole learning led by pure punishment did not transfer to the counter-rotation learning. A possible explanation is that people’s sensitivity to rotational errors, regardless of their sizes or directions, has been largely enhanced when learning accompanied by combining punishment and reward. That is to say, structural learning could possibly be activated specifically through motivation by combining punishment and reward. Structural learning is a meta-learning phenomenon evidenced by an accelerated learning rate for novel tasks sharing the same statistics as the training task [22]. Although the specific solutions to deal with initial rotation learning and counter-rotation learning are to the opposite directions, the structures for both perturbations are the same [23, 24]. The fact that the motivational effect of combining punishment and reward transferred to the opposite-rotation learning again confirms its effectiveness on motor learning.

Motivational effects on memory consolidation defined as reduced fragility of fresh memory [5] has never been tested in motor adaptation. In our study, we failed to find any significant difference among the four groups in terms of memory consolidation. The learning advantage of combining punishment and reward was not retained through the relearning phase after interference by opposite-rotation learning. It has been suggested that consolidation can be triggered by reaching asymptotic or saturated performance within the training session [8, 25]. A recent study shows that long-term motor memory could be enhanced by reward interacting with repetition-dependent learning [26]. However, actions are adjusted little by little over a number of trials when a previously unseen perturbation is applied to movements abruptly [27]. Adaptation learning takes time to reach an asymptote or to become saturated. Similar to the findings in other studies [6, 9], participants still made a sizeable error of about 10 degrees after learning 40 cycles in the initial rotation learning phase. The learning seemed to be still on and did not reach an asymptote. Therefore, the lack of a motivational effect on memory consolidation may be due to the fact that participants were not reinforced extensively during initial learning. We speculate that motivational feedback might work on consolidation if given more practice during initial learning.

The present study did not show a retention difference among the four experimental groups during both no-vision phases. In motor adaptation, researchers have not reached a consensus concerning the motivational effect on immediate retention. Some researchers find reward enhances retention [6, 15, 26], while some find both punishment and reward impair retention [13]. Still, some fail to find any difference made by punishment or reward on retention [9]. In the abovementioned studies, although the retention is measured exclusively immediately after learning when no visual feedback is provided, the differences in the instructions during no-vision phases might partly cause this inconsistency. Motor adaptation has previously been thought to occur by implicitly updating an internal forward model [28, 29]. However, researchers have increasingly realized the existence and importance of the explicit learning component of motor adaptation [30]. Aftereffects during the no-vision phase could reflect memory of pure implicit learning, or a combination of both explicit and implicit learning, depending on the instructions [31, 32]. In the present study, participants were instructed to aim directly to the target and not to use any explicit strategy, excluding the explicit learning component. However, no specific instructions were introduced in other studies. Thus, retention in our study may reflect memory of pure implicit learning while retention in others may include both implicit and explicit components. The lack of groups difference during no-vision phases suggests that memory retention of implicit learning is similar across groups and implicit component may not be influenced by motivational feedback.

As we observed no significant differences in memory retention among groups, and explicit learning component is largely excluded in the measurement, we speculate that the unique learning features of combining punishment and reward are likely to be the contribution of explicit learning component. That is, the explicit learning component is largely enhanced during both initial rotation learning and counter-rotation learning by motivation through combining punishment and reward. This is in accordance with a recent finding suggesting a pivotal role of explicit processes during reinforcement-based motor learning [31, 32]. Making both punishment and reward possible in one phase could maximize the benefits of punishment and reward, promoting both punishment-avoiding and reward-seeking mechanism simultaneously. This explicit reinforcement might enhance people’s sensitivity to directional errors and may promote the formation of structural learning without compromising the implicit remapping processes [22].

Although participants were given veridical cursor feedback, they still showed bias during the late baseline phase. The significant CCW baseline bias could be explained by biomechanical factors observed when people make fast right-handed movements [33, 34]. However, it is surprising that young participants showed a baseline bias of approximately 10° from the very beginning prior to becoming familiar with the virtual environment and task requirements. This may be due to the fact that all four targets were in oblique directions. It is possible that participants would make more directional errors when moving to targets in oblique directions than cardinal ones, as directed perception is generally better in cardinal than oblique directions [35]. Moreover, it is assumed that people’s error sensitivity is higher in cardinal than in oblique directions [35, 36]. Therefore, if the motivational effect of combining punishment and reward results from enhanced sensitivity to directional errors as we speculated above, and the targets are in cardinal directions, the effect could be more salient than we found here.

As mentioned above, researchers have used different tasks and different paradigms to dissociate the different effects of punishment and reward. For motor skill learning, it was found that punishment enhances movement vigor [4], while reward enhances memory retention [5, 37]. However, it was also found that reward has no effect on learning or retention [38]. It has been suggested that the impact of reward and punishment on skill learning depends on task demands. For motor adaptation, researchers seem to have reached an agreement that punishment accelerates learning [6, 9, 14, 16]. However, as mentioned above, although using similar visuomotor rotation tasks, researchers have not reached an agreement about the motivational effect on memory retention. In our experiment, we found that pure punishment accelerates learning rate during initial learning and makes people learn more completely during counter-rotation learning. Pure reward shows no benefit or impairment on learning or memory. Combining punishment and reward shows learning advantage throughout the motivational phase and the effect could even transfer to opposite rotation learning. It reflects the unique learning features of combining punishment and reward, instead of a simple superposition of the effect of punishment and reward. Although interesting, these complicated results make it difficult to draw any firm conclusions regarding the influence of punishment and reward in laboratory-based motor learning [39]. As a matter of fact, in the real world, whether in sports or education, situations in which either punishment or reward is used exclusively are indeed rare. Therefore, it is imperative to examine the combined effect of punishment and reward in different tasks and paradigms.

Overall, we have demonstrated that combining punishment and reward could both accelerate learning rate and increase learning extent during the motivational phase. More interestingly, the effect can even transfer to later opposite-direction learning. The results suggest a distinct effect of combining punishment and reward on learning and memory in motor adaptation task, instead of simply a superposition of the individual effects of either punishment or reward. This distinct motivational effect may result from enhanced sensitivity to directional errors through an explicit learning mechanism.
